# Chlorogenic Acid Decreases Intestinal Permeability and Increases Expression of Intestinal Tight Junction Proteins in Weaned Rats Challenged with LPS

**DOI:** 10.1371/journal.pone.0097815

**Published:** 2014-06-02

**Authors:** Zheng Ruan, Shiqiang Liu, Yan Zhou, Shumei Mi, Gang Liu, Xin Wu, Kang Yao, Houssein Assaad, Zeyuan Deng, Yongqing Hou, Guoyao Wu, Yulong Yin

**Affiliations:** 1 State Key Laboratory of Food Science and Technology, Nanchang University, Nanchang, Jiangxi, China; 2 Institute of Subtropical Agriculture, Chinese Academy of Sciences, Changsha, Hunan, China; 3 Department of Statistics, Texas A&M University, College Station, Texas, United States of America; 4 Hubei Key Laboratory of Animal Nutrition and Feed Science, Wuhan Polytechnic University, Wuhan, Hubei, China; 5 Department of Animal Science, Texas A&M University, College Station, Texas, United States of America; SRI International, United States of America

## Abstract

Chlorogenic acid, a natural phenolic acid present in fruits and plants, provides beneficial effects for human health. The objectives of this study were to investigate whether chlorogenic acid (CHA) could improve the intestinal barrier integrity for weaned rats with lipopolysaccharide (LPS) challenge. Thirty-two weaned male Sprague Dawley rats (21±1 d of age; 62.26±2.73 g) were selected and randomly allotted to four treatments, including weaned rat control, LPS-challenged and chlorogenic acid (CHA) supplemented group (orally 20 mg/kg and 50 mg/kg body). Dietary supplementation with CHA decreased (P<0.05) the concentrations of urea and albumin in the serum, compared to the LPS-challenged group. The levels of IFN-γ and TNF-α were lower (P<0.05) in the jejunal and colon of weaned rats receiving CHA supplementation, in comparison with the control group. CHA supplementation increased (P<0.05) villus height and the ratio of villus height to crypt depth in the jejunal and ileal mucosae under condictions of LPS challenge. CHA supplementation decreased (P<0.05) intestinal permeability, which was indicated by the ratio of lactulose to mannitol and serum DAO activity, when compared to weaned rats with LPS challenge. Immunohistochemical analysis of tight junction proteins revealed that ZO-1 and occludin protein abundances in the jejunum and colon were increased (P<0.05) by CHA supplementation. Additionally, results of immunoblot analysis revealed that the amount of occludin in the colon was also increased (P<0.05) in CHA-supplemented rats. In conclusion, CHA decreases intestinal permeability and increases intestinal expression of tight junction proteins in weaned rats challenged with LPS.

## Introduction

The intestinal barrier plays a major role in maintaining intestinal homeostasis, which is a highly dynamic interface between external foods/microbes and internal environment of the body. It is composed of the apical cell membrane and intercellular tight junctions of enterocytes [1]. When the intestinal barrier is disturbed, it results in translocation of luminal pathogens and antigens into subepithelial tissues or mucosal and systemic inflammation [2]. Therefore, an intact intestinal mucosal barrier is very important in both preventing gut-related diseases and ensuring adequate provision of dietary nutrients to the whole body [3]. In fact, many factors can affect mucosal barrier function, including food, weaning stress, infection, microorganism and inflammation [4], [5]. Early weaning stress can result in damage of internal integrity in animals, which include intestinal disorders and immunocompetence, intestinal barrier disturbances, villous atrophy and crypt hyperplasia [6]. Several studies demonstrated that weaning stress induced impairment in intestinal epithelial barrier function and increased disease susceptibility [7], [8].

Chlorogenic acid (CHA), formed by esterification of caffeic and quinic acids, is one of the most abundant phenolic acid in nature [9]. It is widespread in plants, fruits and vegetables [10]. CHA has a wide range of biological activities and has been shown to exert potent immunoprotective, anti-inflammatory, anti-bacterial and anti-oxidant activities [11], [12]. CHA has been reported to induce human lymphocytes and human peripheral blood leukocytes to produce IFN-γ and IFN-α [13]. Gong et al. [14] suggested that CHA-treated mice enhanced the level of IgE, IgG, and IL-4 in vivo. Although the information about the effect of phenolic acid on intestinal function is quite limited, some papers disclosed that phenolic acids or flavonoids affected intestinal permeability [15]. Studies in vitro showed that ferulic acid increases zonula occludens-1 (ZO-1) and claudin-4 transcription in T84 colon cells [16]. In addition, Suzuki et al. [17] reported that quercetin and myricetin stabilized intestinal barrier function integrity by the maintenance of tight junction protein expression through by inhibiting the PKC-δ isoform in human intestinal Caco-2 cells. Furthermore, recent studies found that CHA regulated the intestinal mucosal immune function, intestinal flora [18] and antioxidant activities against ischemia and reperfusion injury in the rat small intestine [19]. However, little is known about the protective effect and mechanisms of CHA on intestinal permeability and tight junction.

Therefore, we use an animal model to induce intestinal injury in weaned rats. The objective was to evaluate whether CHA could mitigate the impairment of intestinal barrier function in weaned rats.

## Materials and Methods

### Animals, diet and experimental design

Sprague Dawley rats were purchased from Hunan SLAC King of Laboratory Animal Limited Company. All procedures were approved by the Nannchang University Animal Care and Use Committee. The rats were housed individually in a temperature-controlled room with a 12 h light/12 h dark cycle. The experimental diets were formulated to meet the nutrient requirements (Table 1) [20]. A total of 32 male rats weaned at 21±1 d of age (initial BW = 62.26±2.73 g) were used in this experiment. The rats were randomly assigned to one of the four groups (n = 8). Four treatments were as follows: (1) weaned rats (Control group), fed the control diet and orally administrated with sterile saline; (2) weaned rats challenged with LPS (LPS group), rats fed the same control diet and intraperitoneally injected with LPS (1 mg/Kg body weight) at 13th day after weaning; (3) 20 mg/kg CHA+LPS (CHA20), rats fed the same control diet, orally daily administrated with 20 mg/kg CHA in 14 days experimental period and intraperitoneally injected with LPS (1 mg/Kg body weight) at 13th day after weaning; (4) 50 mg/kg CHA+LPS (CHA50), rats fed the same control diet and orally daily administrated with 50 mg/kg CHA in 14 days and intraperitoneally injected with LPS (1 mg/Kg body weight) at 13th day. The rats were sacrificed on the 15^th^ day after weaning.

**Table 1:** The composition and nutrient of experiment diet*.

Ingredients	Content (%)	Chemical composition	Content
Wheat	14	Digestible energy (Mal/kg)	3.40
Corn	43	Crude protein (%)	21.0
Soybean meal	24	Crude Fat (%)	4.5
Full fat soybean extruded	8	Calcium (%)	1.0
Soybean oil	1.4	Total phosphate (%)	0.7
Whey powder	3		
Fish meal	3.2	Sodium (%)	0.3
Limestone	1.3	Met+Cys (%)	0.78
Dicalcium phosphate	1.1	Lys (%)	1.35
Vitamin-mineral premix#	1.0	Thr (%)	0.88

*The nutrient levels of the diets were based on China General Quality Standards for Animal Feed (GB14924.1-2001).

# The vitamin-mineral premix provided (per kilogram feed): vitamin A, 14,000 IU; vitamin D_3_, 1500 IU; vitamin E, 5 mg; vitamin K, 5 mg; thiamine, 13 mg; riboflavin, 12 mg; pyridoxine, 12 mg; vitamin B_12_, 0.022 mg; niacin 60 mg; pantothenic acid, 24 mg; biotin, 0.2 mg; folic acid, 6 mg; choline chloride, 350 mg; Fe (as iron sulfate), 120 mg; Cu (as copper oxide), 10 mg; Mn (as manganous oxide), 75 mg; Zn (as zinc oxide), 30 mg; I (as ethylenediamine dihydroiodide), 0.5 mg; and Se (as sodium selenite), 0.2 mg.

### Measurements and sample preparation

Throughout the study, feed intake and body weight of the rats were recorded daily. The feed to gain ratio were calculated.

After the rats were euthanized [9], the intestine samples were harvested. Blood samples were allowed to clot at 4°C and centrifuged at 4000×g for 10 min to obtain serum. The serum samples were stored at −20°C until they were analyzed. Gut tissue samples (jejunum, ileum and colon) from each group were collected and frozen in liquid nitrogen and then stored at −80°C until analysis. Each segment (approximately 2 cm length, respectively) was flushed with a 0.9% salt solution, fixed with 10% formaldehyde-phosphate buffer, and kept at 4°C for a microscopic assessment of the mucosal morphology and immunohistochemistry analysis.

### Serum biochemical parameters

The activities of the serum total protein (TP), urea nitrogen (BUN), glucose (GLU) and albumin (ALB) were determined using an Automatic Biochemistry analyzer (CX4, Beckman, Coulter, Brea, CA) [21]. Test kits were purchased from Beijing Leadman Biochemistry Technology Company (Beijing, China).

### Measurement of inflammatory cytokines

Cytokines were measured using commercial ELISA kits (IL-10, Boster, Wu han, China; TNF-α and IFN-γ, Shanghai Xin Yue Biotechnology, Shanghai, China) according to the published methods [22]. All analyses were conducted as described by the manufacturer.

### Small intestinal morphology

Formalin-fixed intestinal samples were prepared using paraffin embedding techniques. Samples were sectioned at a 5-µm thickness and stained with hematoxylin and eosin. The method was the same as described by Nabuurs et al method [23]. Photomicrographs were acquired with 100× magnifications using an Olympus BX51 microscope (Olympus Optical Company, Shanghai, China). Villous height and the associated crypt depth were evaluated using the Image-Pro Plus 6.0 image processing and analysis system. For each intestinal sample, at least 10 well-oriented were measured and the mean value was calculated.

### Serum diamine oxidase activity

The serum diamine oxidase (DAO) activity was determined according to the method [24]. Blood samples were centrifuged at 4000×g for 10 minutes at 4°C. The sera were kept at −80°C until measurement. The DAO activity was determined using automatic biochemistry analyzer (Beckman, CX4, CA, USA). The assay kit was purchased from the Nanjing Jiancheng Bioengineering Institute, (Nanjing, China).

### Lactulose-mannitol test

Intestinal permeability was assessed by the lactulose-mannitol test [25]. The test solution consisted of 100 mg of lactulose (Sigma-Aldrich, Tokyo, Japan) and 50 mg of mannitol (Sigma-Aldrich), which were mixed in 2 mL of physiologic saline. The test solution was administered orally. All the urine was collected for 6 h and mixed thoroughly. A 2-mL sample was taken from the pooled urine and frozen at −20°C until analysis. Urinary lactulose and mannitol concentrations were measured by high-performance liquid chromatography (HPLC).

### Immunohistochemistry analysis for occludin and ZO-1

Rats jejunum and colon samples were removed and fixed in 10% buffered formalin, embedded in paraffin and cut into 5 µm-thick sections [26]. Briefly, sections were deparaffinized and washed in PBS, soaked in 3% H_2_O_2_ for 10 min, and then antigen retrieval, washed in PBS, and incubated with goat serum albumin for 20 min. Sections were then incubated with rabbit anti-occludin (1∶100, Invitrogen, Carlsbad, CA, USA) or rabbit anti-zonulin-1 (1∶100, BS-1329R, Bios, Beijing, China) at 37°C for 2.5 h. The embedded tissues were washed with PBS. After the sections were incubated with biotinylated anti-rabbit IgG and then processed by the S-A/HRP, color was developed in the diaminobenzidine (DAB) substrate solution. The sections were then counterstained with hematoxylin, dehydrated, cleared, and permanently mounted. The sections were observed under the Olympus BX51 microscope (Olympus Optical Company, Shanghai, China).

### Western blot analysis for occludin

Occludin protein was measured by the western blotting technique [27]. Colon tissue specimens obtained from four groups were frozen in liquid nitrogen. Frozen tissue samples were washed with PBS. The samples were then lysed on ice in a Potter tissue grinder with lysis buffer (20 mM Tris-HCl, pH 8.0, 5 mM EDTA, 1% Triton ×100) supplemented with a protease inhibitor cocktail (Cat. 539134, Merck, Darmstadt, Germany). Each tissue was homogenized and then sonicated for three times (20 s each). The homogenate was centrifuged at 10,000×g for 5 min at 4°C. Quantification of protein in the supernatant fluid was determined using the bicinchoninic acid (BCA) protein assay by the microplate procedure. Total proteins (30 µg) from each tissue was separated by SDS-PAGE and transferred onto PVDF membranes. The blots were blocked for 30 min in 5% non-fat milk in phosphate-buffered saline before incubation with primary antibodies and overnight in 5% non-fat milk in PBS at 4°C. After thorough washing, the membranes in 5% non-fat milk in PBS were incubated with rabbit anti-occludin (invitrogen, 71–1500 USA) and goats anti-β-actin (BS-0061R, Bios, Fir Jinqiao Beijing, China) antibodies diluted 1∶3,500 and 1∶1000 in 5% skim milk PBS-Tween-SBA respectively. After washing, they were incubated with 1∶5,000 horseradish peroxidase-conjugated anti-rabbilt IgG. After thorough washing, the Superstar Enhanced chemiluminescent kit (Pierce Ultrasensitive kit AR1111, Boster, Wuhan, China) was applied for antibody detection with chemiluminescence in order to rebind another protein of interest in the same membrane.

### Statistical analysis

All data were statistically analyzed using one-way ANOVA and the Tukey multiple comparison test using the statistical package of SPSS 17.0 (SPSS Inc., Chicago, USA). Results are expressed as mean ± SE. Probability values ≤0.05 were taken to indicate statistical significance.

## Results

### Growth performance

The growth performance of rats without LPS challenge is shown in Table 2. From d 1 to 7 and d 7 to 14 post-weaning, rats fed CHA (20 mg/kg or 50 mg/kg) had similar (P>0.05) ADG and ADFI, compared with control rats. From d 1 to 7 after weaning, there were no significant differences in the feed:gain ratio among the 3 treatments. However, rats fed CHA (50 mg/kg) had a lower feed:gain ratio (P<0.05) compared with control rats. This result indicated that CHA promoted nutrient utilization or increased nutrient bioavailability.

**Table 2:** Effects of CHA on growth performance of weaned rats.

Item	Period	Control		LPS		CHA20		CHA50	
		Mean	SE	Mean	SE	Mean	SE	Mean	SE
ADG (g)	d 1 to 7	3.80	0.18	3.78	0.19	4.01	0.16	3.88	0.14
	d 7 to 13	7.32	0.32	7.33	0.34	7.52	0.35	8.35	0.37
	d 7 to 14	7.72	0.35	7.52	0.36	7.94	0.32	8.66	0.31
ADFI (g)	d 1 to 7	18.01	0.58	17.95	0.55	17.78	0.32	16.73	0.59
	d 7 to 13	17.73	0.53	17.65	0.51	17.51	0.41	18.08	0.43
	d 7 to 14	17.97	0.56	17.75	0.53	17.73	0.43	18.28	0.47
F:G (g/g)	d 1 to 7	4.57	0.20	4.55	0.22	4.28	0.18	4.31	0.22
	d 7 to 13	2.78^b^	0.18	2.79^b^	0.18	2.51^a^	0.15	2.42^a^	0.14
	d 7 to 14	2.53^b^	0.15	2.58^b^	0.17	2.27^a^	0.14	2.05^a^	0.10

ADFI, Average daily feed intake; ADG, Average daily gain; F:G, Feed:Gain; CHA, chlorogenic acid.

At 13th day after weaning, the rats (LPS group and CHA groups) received intraperitoneal administration of LPS.

CHA20, rats supplemented with 20 mg/kg chlorogenic acid; CHA50, rats supplemented with 50 mg/kg chlorogenic acid.

a,b Means in the same row with different superscripts differ (P<0.05).

### Serum biochemical parameters

The concentration of BUN in the serum of CHA-supplemented rats was decreased (P<0.05), compared to rats challenged with LPS (Table 3). Rats in the CHA20 and CHA50 groups had a lower (P<0.05) concentration of ALB, compared to the LPS group. However, there were no significant differences (P>0.05) in TP or GLU among the 4 groups of rats.

**Table 3:** Effects of CHA on serum biochemical parameters in weaned rats with LPS challenge.

Item	Control		LPS		CHA20		CHA50	
	Mean	SE	Mean	SE	Mean	SE	Mean	SE
BUN (mmol/L)	5.88^ab^	0.13	7.18^c^	0.34	6.49^b^	0.16	5.29^a^	0.18
TP (g/L)	60.9	1.5	62.3	2.4	61.0	2.3	57.0	1.6
ALB (g/L)	32.8^b^	1.0	36.2^c^	1.2	30.7^ab^	1.1	28.2^a^	0.7
GLU (mmol/L)	4.88	0.21	4.65	0.19	5.08	0.24	5.23	0.21

CHA, chlorogenic acid; BUN, urea nitrogen; TP, total protein; ALB, albumin; GLU, glucose.

Control, diet with sterile saline; LPS, control rats injected with LPS at d 13; CHA20, rats supplemented with 20 mg/kg every day and challenged with LPS at d 13; CHA50, rats supplemented with 50 mg/kg every day and challenged with LPS at d 13.

a,b,c Means in the same row with different superscripts differ (P<0.05).

### Intestinal inflammation

**Figure 1:**
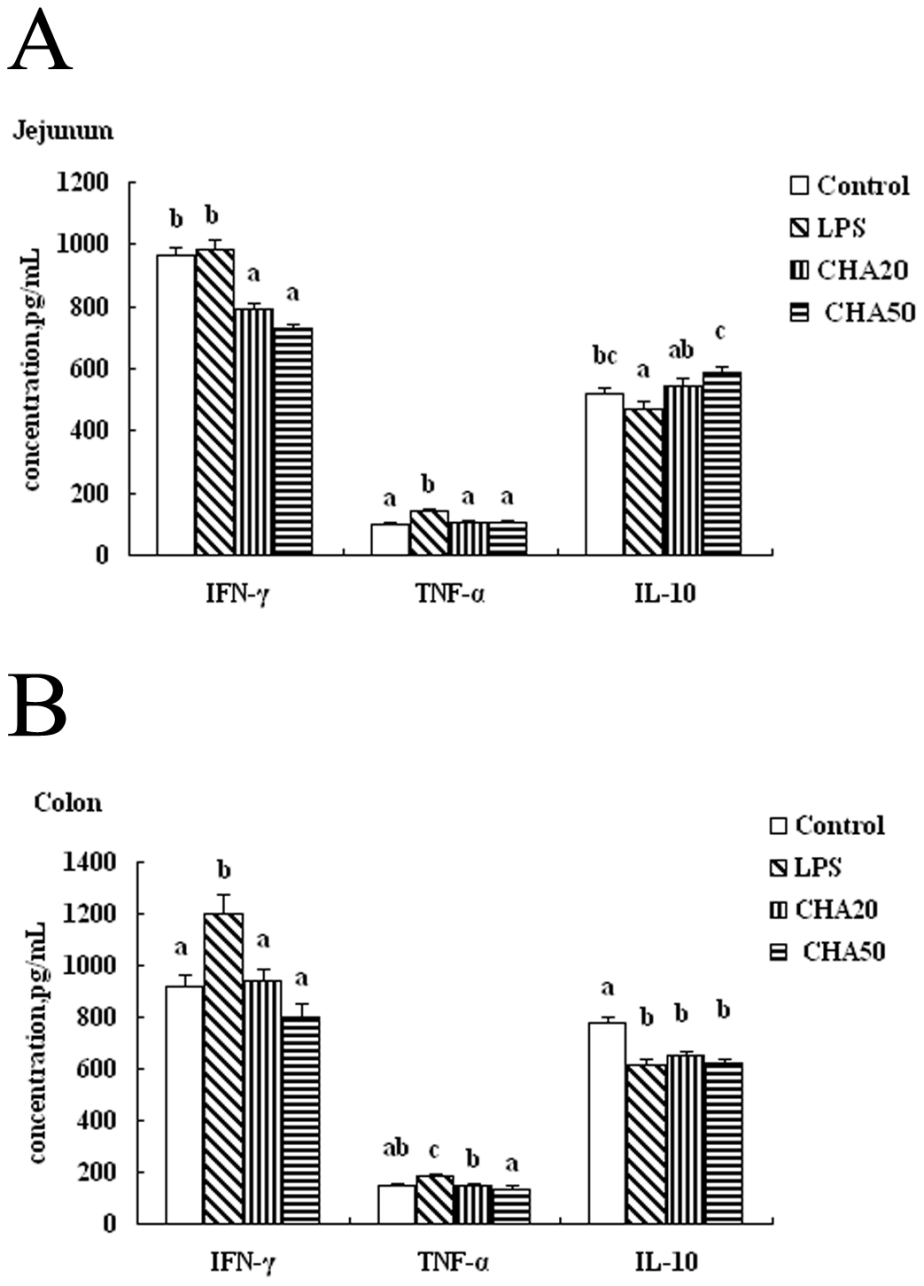
Effects of CHA on IFN-γ, TNF-α and IL-10 in the jejunum and colon of weaned rats. Control, rats fed the control diet and administered with sterile saline; LPS, control rats injected with LPS at d 13; CHA20, rats supplemented with 20 mg/kg every day and challenged with LPS at d 13; CHA50, rats supplemented with 50 mg/kg every day and challenged with LPS at d 13. ^a,b,c^Means in the same row with different superscripts differ (P<0.05).

To gain insight into the effect of CHA on inflammatory cytokines in the intestinal tissue of weaned rats, we determined the concentrations of IFN-γ, TNF-α and IL-10 in the jejunum and colon. CHA50 and CHA20 significantly reduced (P<0.05) the concentration of IFN-γ in the jejunum and colon, compared with weaned rats challenged with LPS (the LPS group) (Fig. 1A). Rats in the CHA 20 and 50 groups had a lower (P<0.05) concentration of TNF-α, compared to the LPS group (P<0.05). Only CHA 50 affected (P<0.05) the concentration of IL-10 I in the jejunum, compared to the LPS group (Fig. 1B).

### Intestinal morphology analysis

**Figure 2:**
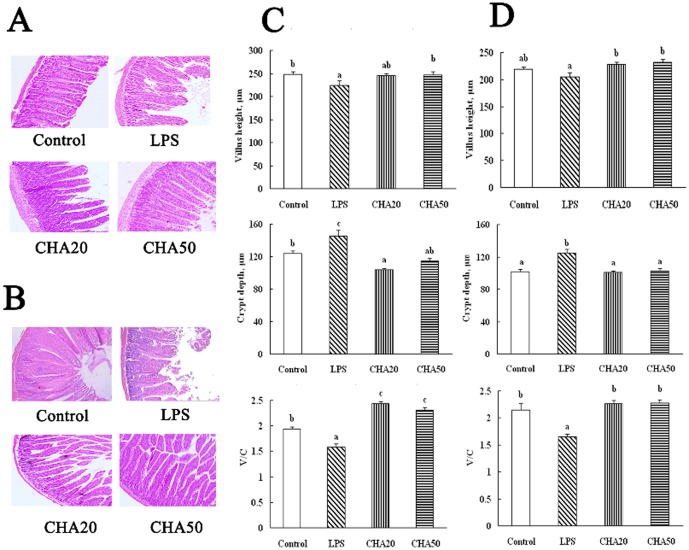
Effects of CHA on villus height, crypt depth and ratio of villous height to crypt depth in the jejunum and ileum of weaned rats. A, representative figures of histological sections of the ileum. B, representative figures of histological sections of the jejunum. C, villus height, crypt depth, and the ratio of villous height to crypt depth of the ileum in weaned rats. D, villus height, crypt depth, and the ratio of villous height to crypt depth of the jejunum in weaned rats. Hematoxylin and eosin stain, 200X. Significant differences (P<0.05) are identified by different letters, a–c.

After LPS was administered to rats, CHA 50 significantly increased (P<0.05) villus height in the ileum, compared to weaned rats challenged with LPS (the LPS group) (Fig. 2B), and feeding CHA 20 and CHA 50 significantly increased (P<0.05) villus height in the jejunum (Fig. 2D). The crypt depth was lower (P<0.05) in the jejunum and ileum in CHA-supplemented rats than in the LPS group (P<0.05). The ratio of villus height to crypt depth in the jejunum and ileum was higher (P<0.05) in the CHA and control groups, compared to the LPS group (P<0.05).

### Intestinal permeability

**Figure 3:**
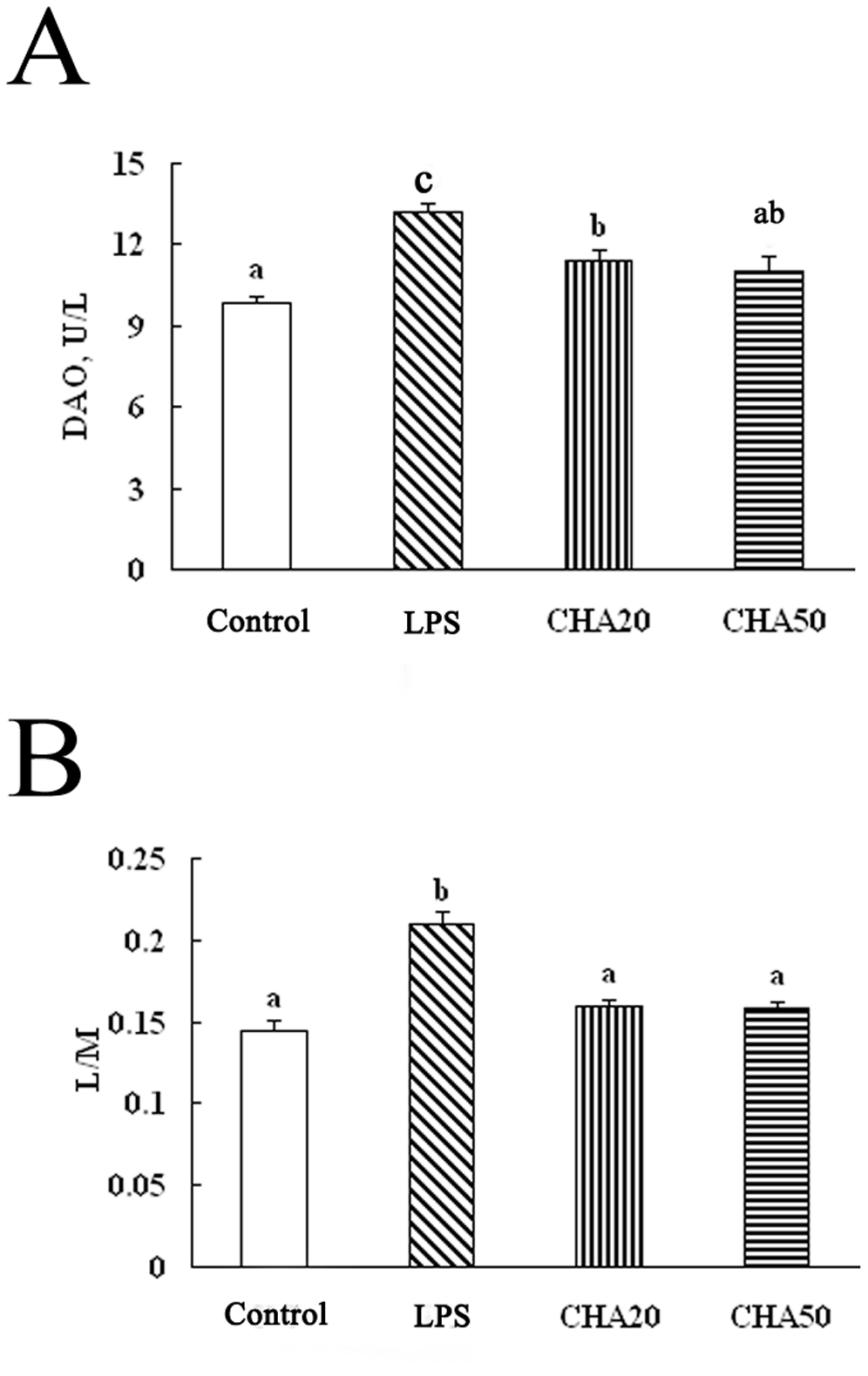
Effects of CHA on intestinal permeability of weaned rats with LPS challenge. Significant differences (P<0.05) are identified by different letters, a, b and c. CHA, chlorogenic acid; DAO, diamine oxidase; L/M, concentration of lactulose/concentration of mannitol.

The results on DAO activity in the serum and the ratio of lactulose to mannitol (L/M) in the urine are shown in Fig. 3. After LPS challenge, weaned rats receiving LPS challenge (the LPS group) had a higher (P<0.05) activity of DAO in the serum and a high L/M ratio in the urine, compared to the control group. These data indicated that the weaned rats with the LPS treatment exhibited an increase in intestinal permeability. Oral supplementation with CHA reduced (P<0.05) DAO activity in the serum and the ratio of L/M in the urine, compared to the LPS group. This result indicated that CHA enhanced intestinal integrity. In addition, the correlation coefficient between DAO activity and the L/M in the urine is 0.931 (y = 0.0184x−0.0437, R = 0.931).

### Immunohistochemical analysis of intestinal occludin and ZO-1 proteins

**Figure 4:**
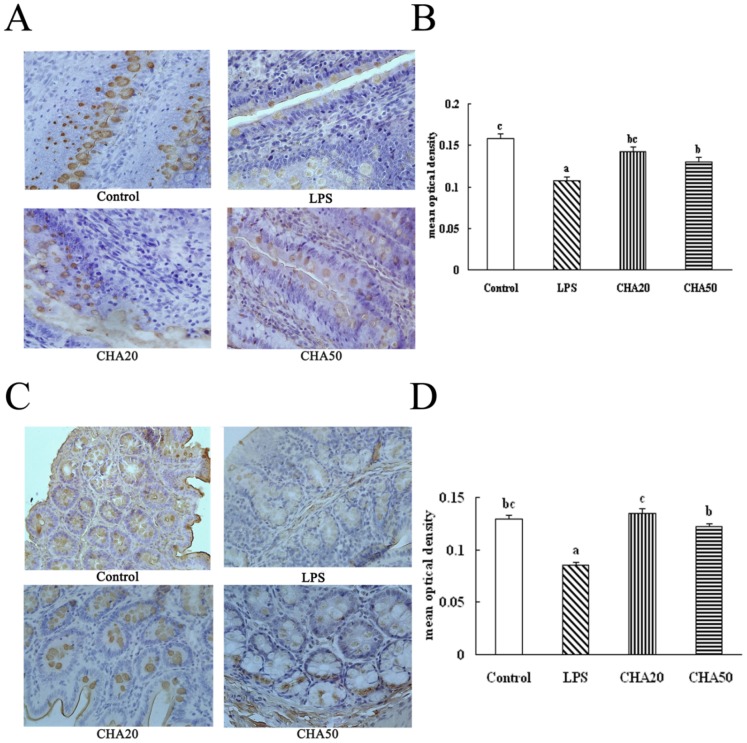
Effects of CHA on occludin in the jejunum and colon of weaned rats. Occludin protein in intestinal tissue was measured by immunohistochemical methods. Occludin positive cells were stained in the brown color and present on the cell membrane. The bigger brown and deeper color represents the higher occludin protein level. A, representative figures of occludin in the jejunum. B, mean optical density of occludin in the jejunum. C, representative figures of occludin in the colon. D, mean optical density of occludin in the colon. All pictures are shown at the 400× magnification. Significant differences (P<0.05) are identified by different letters a, b and c.

**Figure 5:**
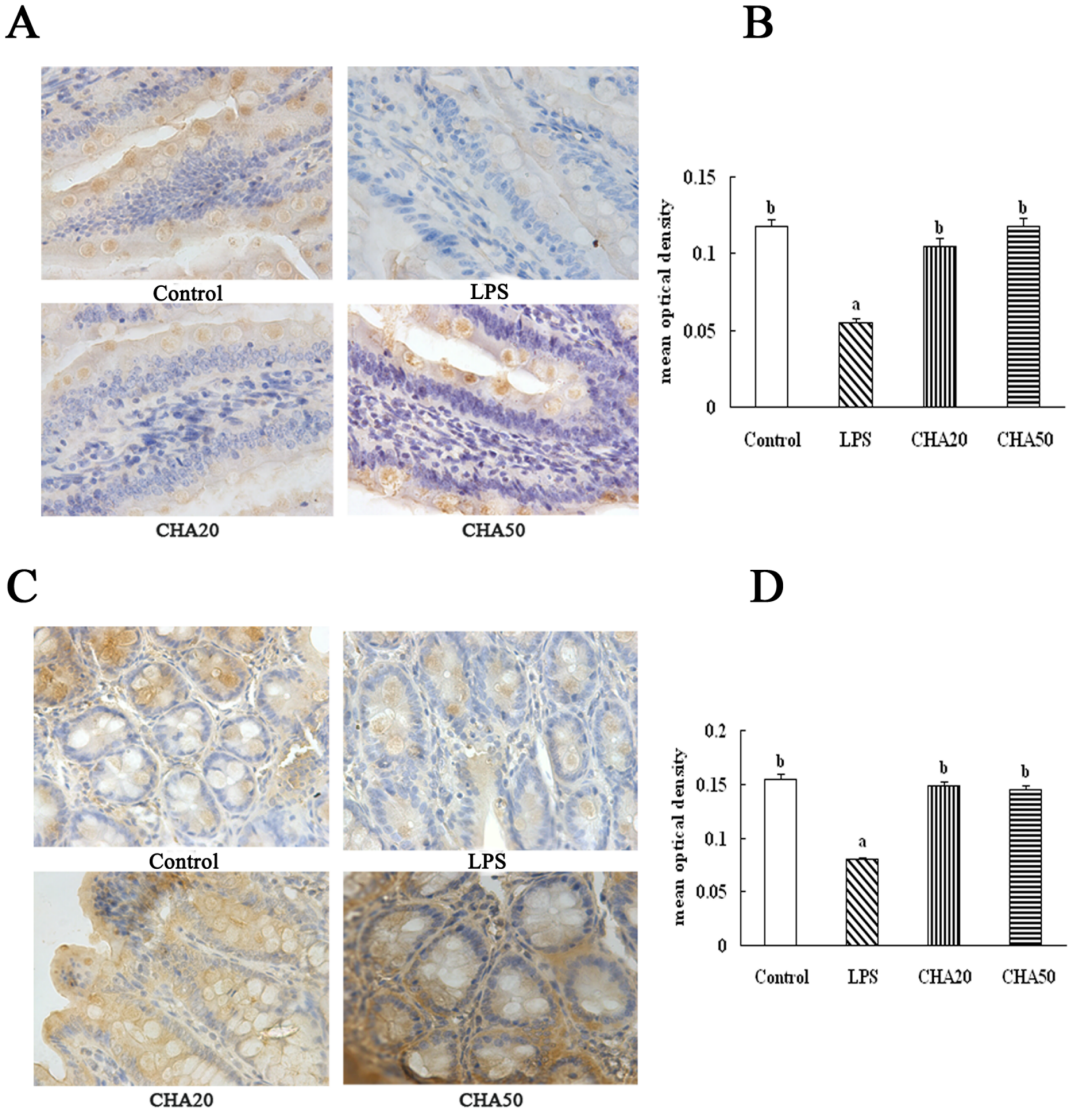
Effects of CHA on ZO-1 in the jejunum and colon of weaned rats. ZO-1 protein in intestinal tissues was measured by immunohistochemical method. ZO-1 positive cells were stained in brown and present on the cell membrane. The bigger brown and deeper color represents the higher ZO-1 protein level. A, representative figures of ZO-1 in the jejunum. B, mean optical density of ZO-1 in the jejunum. C, representative figures of ZO-1 in the colon. D, mean optical density of ZO-1 in the colon. All pictures are shown at the 400× magnification. Significant differences (P<0.05) are identified by different letters a, b and c.

The LPS challenge decreased (P<0.05) the abundances of occludin and ZO-1 in the jejunum and colon, compared to the control group (Fig. 4, 5). Oral supplement with CHA increased (P<0.05) the protein levels for occludin and ZO-1 in the jejunum and colon, compared to the weaned rats challenged with LPS (the LPS group) (P<0.05). These results indicated CHA supplementation increased the tigh junction in the jejunum and colon of weaned rats.

### Western blot analysis for occludin

**Figure 6:**
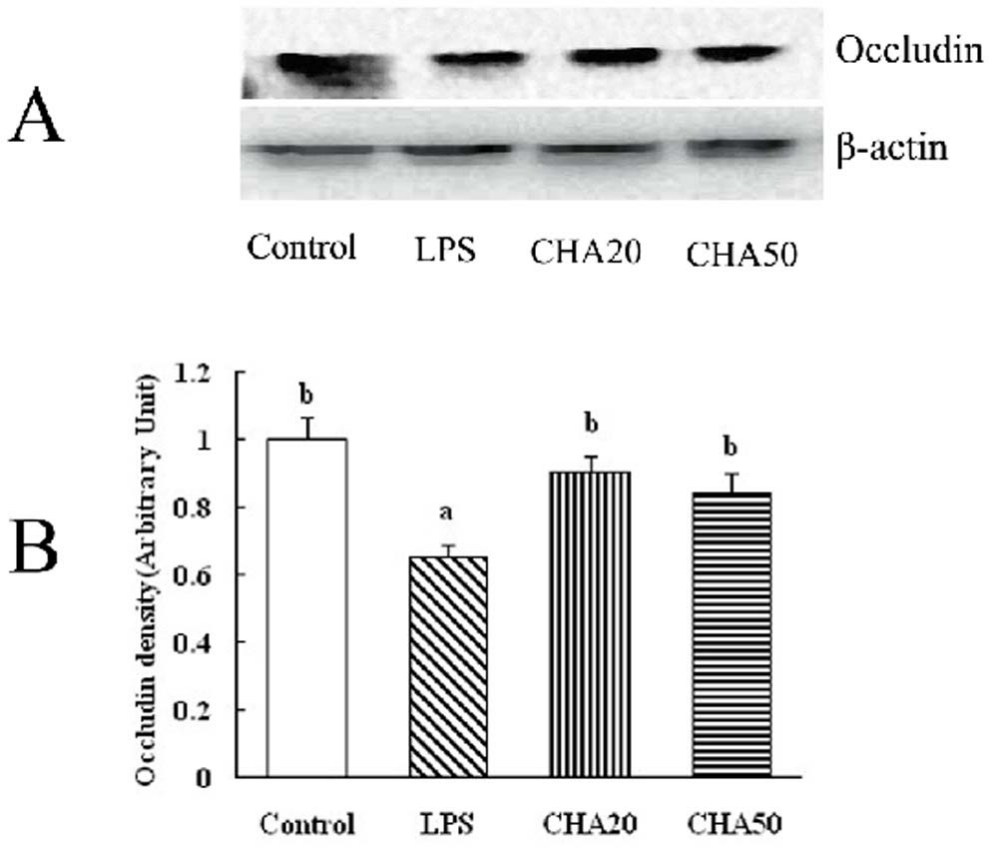
Effects of CHA on occludin protein in the colon of weaned rats with western blot. Mucosal extracts (120 µg protein/sample) were separated by 10% SDS–polyacrylamide gel electrophoresis for determination of occludin and β-actin. A, representative immunoblots. Each specific band of proteins was quantitated by densitometric analysis. B, relative density values. Significant differences (P<0.05) are identified by different letters a and b.

LPS administration decreased (P<0.05) the abundance of the occludin protein in the colon mucosa, compared to the control group. Oral supplementation with CHA increased (P<0.05) occludin expression in the colon mucosa, compared to the LPS group (Fig. 6).

## Discussion

This present investigation is one of the first studies testing the hypothesis that CHA can decrease the intestinal permeability and increase expression of the intestinal tight junction proteins. Impaired intestinal permeability in weaned animals or infants is associated with enteropathy, infection, and growth faltering. Intestinal permeability can be commonly assessed by many indices, such as DAO activity [28], D-lactic acid concentration [29], or the ratio of concentration of lactulose vs mannitol in urine [30]. DAO, as a relatively stable marker of maturation and integrity of intestinal mucosa cells, is an intracellular enzyme synthesized primarily in the gastrointestinal mucosal cells of mammalian species and distributed primarily in the cytoplasm [31]. The activity of DAO in serum increases when the epithelium is injured and, therefore, DAO activity in serum can reflect the changes in intestinal-mucosa integrity and barrier function [32]. In the current study,LPS treatment increased the serum DAO concentration, which indicates that LPS damages the intestinal barrier. Oral CHA administration decreased DAO activity in serum compared to LPS-challenged rats (Fig. 3). In addition, lactulose traverses the intestinal wall by paracellular pathways via the intercellular tight junctions of epithelial crypts, whereas mannitol passes predominantly by the transcellular pathways of epithelial villi [30]. Mannitol is easily absorbed and serves as a marker of transcellular uptake, while lactulose is only slightly absorbed and serves as a marker for mucosal integrity [33]. Thus, the L/M ratio in the urine can reflect the changes of intestinal permeability. In our study, dietary supplementation with CHA decreased the L/M ratio in urine, compared to the LPS rats. These results indicate that CHA supplementation alleviated the LPS-induced injury of the intestinal barrier.

Intestinal permeability is associated with intestinal inflammation [1], [2]. We investigated whether CHA supplementation could ameliorate inflammation in weaned rats with LPS challenge. Results of many studies suggest that the intestinal ischemia/reperfusion injury, LPS challenge, and intestinal inflammatory diseases can induce the expression of pro-inflammatory cytokines in the intestine of humans and animals [4], [34]. Both in vitro and in vivo investigations show that over-production of pro-inflammatory cytokines can have a negative influence on intestinal mucosal integrity, permeability and epithelial functions [35]. For example, Peace et al. [36] demonstrated that early-weaning stress increased the level of TNF-α in colon and IFN-γ in the ileum and colon at 7 d and 14 d. Shi et al. [37] reported that CHA reduced liver inflammation and fibrosis through inhibition of toll-like receptor 4 signaling pathway. Similarly, CHA alleviates ischemia and reperfusion induced liver injury [38]. In the current experiment, we employed LPS as an inflammatory agent to establish a model of gut injury in weaned rats. LPS challenge increased the level of IFN-γ and TNF-α in the jejunum and colon (Fig. 1). Importantly, CHA supplementation reduced the concentrations of IFN-γ and TNF-α in the jejunum and colon, compared to LPS-challenged rats. These findings indicate that the CHA has beneficial effects in reducing intestinal mucosal inflammation.

Because it is unknown how chlorogenic acid exerts its beneficial effect on intestinal barrier integrity, we aimed to investigate whether the CHA supplement might affect expression of intestinal tight proteins. The intercellular tight junction proteins play a major role in maintaining the intestinal barrier function, which are positioned around the apical end of the lateral cell membrane. Tight junction proteins are composed of several transmembrane proteins, such as occludin, and intracellular molecules, such as zonula occludens-1 (ZO-1) [39]. Tight junction proteins are essential for the integrity of the intestinal barrier by building the most apical structure and regulating paracellular permeability and polarity of the cell [40]. Occludin was identified as the first transmembrane protein that both regulates and organizes the tight junction structure [41]. Reducing the protein expression of occludin increases the epithelial permeability in many cell systems [42]. The ZO-1 as one of the most important tight junction proteins, may act as a link between cytoskeleton and other tight junction proteins, which is localized with the cytoplasmic end of occludin at tight junctions [43]. Suzuki et al. [17] found that kaempferol promoted the actin cytoskeletal association of the TJ proteins, ZO-1, ZO-2, occludin and claudin. Zhao et al. [44] demonstrated that the increase of ZO-1 expression plays an essential role in decreasing paracellular permeability. Apple polyphenols and their intestinal metabolites appeared to enhance the epithelial barrier functions in the T84 colonic cell monolayer model [45]. In the current study, we found that LPS challenge decreased the protein level of occludin and ZO-1 in the intestinal mucosa. Notably, CHA supplementation increased ZO-1 and occludin expression in the colon and jejunum mucosa, compared to LPS-challenged rats (Fig. 4, 5, 6).

Corroborating the molecular data on intestinal protein expression, we determined whether the CHA supplement may affect intestinal structure and metabolic function. In the study, chlorogenic acid had a development-promoting effect in the jejunum and ileum, as indicated by changes in serum metabolites (Table 3) and villus structure (Fig. 2), and increased bioavailability of dietary nutrients (Table 2). For example, enhanced absorption and utilization of dietary amino acids for protein synthesis is expected to result in an increase in the concentration of albumin but a decrease in the concentration of urea in the serum [46]–[49]. Villus height, crypt depth and the ratio of villus height to crypt depth can be regarded as a criterion to reflect the intestinal mucosal morphology and the absorption capacity of the small intestine [50]. Thus, an increase in villus height, villus/crypt ratio or decrease in the crypt depth corresponds to an improvement in the digestion and absorption of nutrients [51]–[54]. Accordingly, CHA supplementation increased villus/crypt ratio and villus height in the jejunum and ileum, and decreased the crypt depth, compared to the LPS rats. The result of serum metabolites (Table 3) was also in agreement with the alteration of intestinal villus structure. Based on these results, we concluded that CHA supplementation protected the intestinal mucosa from the LPS-induced cell injury.

In conclusion, results of the present study indicate that dietary supplementation with CHA improves intestinal structure and metabolic function in LPS-challenged weaned rats. CHA also decreases intestinal mucosal damage and enhanced intestinal-mucosal integrity. Furthermore, CHA attenuates the dysfunction of intestinal epithelial tight junction through increasing the abundances of intestinal occludin and ZO-1 proteins in weaned rats with LPS challenge.
